# The Interlinked Rising Epidemic of Insufficient Sleep and Diabetes Mellitus

**DOI:** 10.3390/healthcare7010037

**Published:** 2019-03-05

**Authors:** Vijay Kumar Chattu, Soosanna Kumary Chattu, Deepa Burman, David Warren Spence, Seithikurippu R. Pandi-Perumal

**Affiliations:** 1 Faculty of Medical Sciences, The University of the West Indies, St. Augustine, Trinidad and Tobago; susanna.poul@gmail.com; 2 Global Institute of Public Health, Thiruvananthapuram, Kerala 695024, India; 3 School of Medicine, University of Pittsburgh, 4200 Fifth Ave, Pittsburgh, PA 15260, USA; dr.deepa.burman@gmail.com; 4 Independent Researcher, 652 Dufferin Street, Toronto, ON M6K 2B4, Canada; dwspence@fastmail.fm; 5 Somnogen Canada Inc., College Street, Toronto, ON M1H 1C5, Canada; pandiperumal2019@gmail.com

**Keywords:** diabetes mellitus, insufficient sleep, insufficient sleep syndrome, metabolic syndrome, obesity, obstructive sleep apnea, sleep-disordered breathing

## Abstract

For healthy existence, humans need to spend one-third of their time sleeping. Any qualitative or quantitative disturbances in sleep would result in an increased prevalence of obesity, metabolic disorders, diabetes, cardiovascular diseases, and hypertension. The paper aims to highlight the growing global problem of insufficient sleep and its significant impact on the rising incidence of diabetes mellitus. An extensive literature search was done in all major databases for “insufficient sleep” and “Diabetes Mellitus” for this review. Shorter (<6 h) and longer (>9 h) durations of sleep have been adversely related to insulin resistance. Though the relation between insufficient sleep and diabetes mellitus is more or less understood, little is known about how oversleeping or hypersomnia (10–12 h) increases the risk of diabetes. The relationship between sleep disturbances and diabetes is dual-sided, as chronic sleep disturbances would elevate the risk of developing insulin resistance, while diabetes would worsen the quality of sleep. Both the qualitative and quantitative disturbances in sleep significantly increase the risk of developing diabetes, which is supported by numerous community-based and hospital-based epidemiological studies discussed in this review. Obstructive sleep apnea is one of the most common sleep disorders and is characterized by chronic intermittent hypoxia and increased sympathetic activity, thus leading to a higher prevalence of diabetes. Sleep therapy may serve as a low-cost method for fighting against the rising epidemic of diabetes.

## 1. Introduction

A sleep disorder is usually a medically diagnosed condition (e.g., obstructive sleep apnea, insomnia, restless leg syndrome), not necessarily poor sleep efficiency or short sleep duration. Some people may sleep more, others may sleep less, and some may have difficulty with getting asleep or may be woken up by breathing difficulties while asleep. Thus, a sleep disorder results in changes in the quantity, quality, timing, and duration of nocturnal sleep, along with impaired daytime functioning. Lack of enough sleep (insomnia) and obstructive sleep apnea (OSA) are the two most common sleep disorders. Short sleep duration is not a sleep disorder (some may not get enough sleep by choice and/or may not need a lot of sleep). It is recommended that adults must sleep seven or more hours a day [1]. However, sleep duration during the last 40 years has decreased by two hours due to workload, lifestyle, social activities, and technology [2]. The Centers for Disease Control and Prevention (CDC) data indicate that almost 35% of adults in the US are not sleeping enough, with not much gender and ethnicity difference. The risk of obesity, type 2 diabetes mellitus (T2DM), hypertension, heart attack, coronary heart disease (CHD), stroke, asthma, chronic obstructive pulmonary disease (COPD), cancer, arthritis, depression, and chronic kidney disease (CKD) increases due to lack of sleep [3].

At the same time, OSA is estimated to affect 14–49% of the adult population [4,5]; moderate to severe forms of sleep apnea are present in around 10% of adults above 30 years of age, and its prevalence increases sharply with age. OSA is more common among men than in women [6]. The prevalence of diabetes mellitus is equally common in the US. Latest statistics indicate that more than 100 million US adults have either diabetes or pre-diabetes, among them 30 million (close to 10% of the adult population) have been diagnosed with diabetes [3,7]. Hypoxia also leads to development of insulin resistance by disturbing one’s normal metabolism, which results in the elevated formation of reactive oxygen, which damages various cells. Sleep disturbances include all disorders of initiating and maintaining sleep (DIMS) such as insomnia, disorders of excessive somnolence (DOES), disorders of sleep–wake schedule, and dysfunctions associated with sleep, sleep stages, or partial arousals (parasomnias). Thus, T2DM is one of the most prevalent diseases in the US along with various sleep disorders. These sleep disturbances are inherently linked to the increase of various non-communicable diseases (NCDs), including diabetes [5,8,9,10,11]. Thus, this review aims to address the aspects of insufficient sleep, diabetes mellitus, and their mutual interactions and interlinkages. The main objectives are to address the role of insufficient sleep in the development of diabetes and also to discuss the prevalence of sleep disorders in individuals living with diabetes.

## 2. Materials and Methods

**Figure 1:**
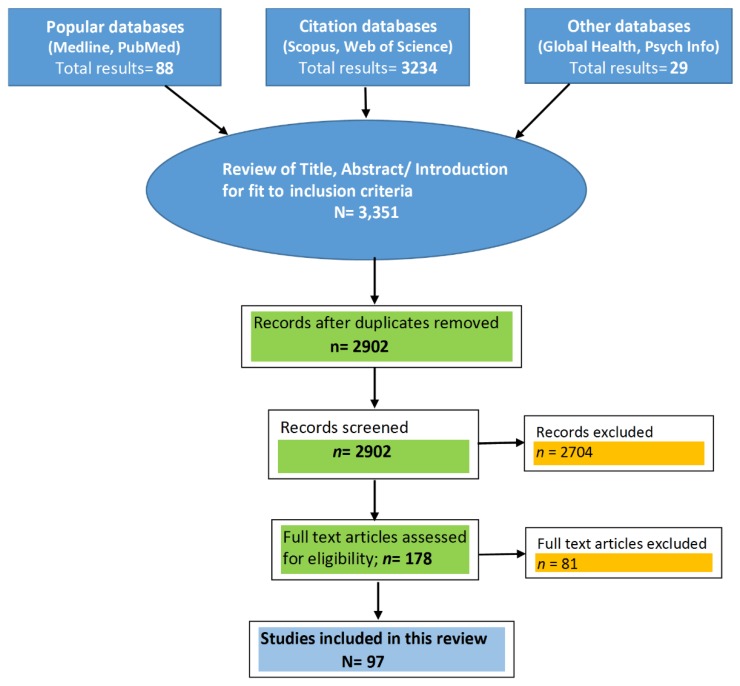
Flow chart showing the literature search.

An extensive literature search was done, and relevant articles were identified by online searches in PubMed, Medline, PsycINFO, Web of Science, Scopus, and Global Health databases. The initial search was done for the keywords ‘Insufficient sleep’ and ‘impacts’. The search was filtered using some inclusion criteria, such as studies being included if they were in English, original or review articles, and focused on Public health/health impacts of insufficient sleep. The selected articles were screened for diabetes mellitus or chronic diseases/non-communicable diseases. The reference lists of the selected articles were checked for additional sources. In this search, around 3351 articles were screened for duplication and relevance to fit the inclusion criteria (“insufficient sleep and diabetes” and “prevalence of sleep disorders among people with diabetes”), and, finally, 97 studies with full texts were included as per the relevance in this review, as shown in Figure 1 below. Articles on cardio-metabolic disorders, OSA, sleep deprivation, sleep restriction, sleep apnea, and obesity were also included to cover the spectrum of insufficient sleep. Valuable information from the various meta-analyses, systematic reviews, longitudinal studies, and cross-sectional studies was referred to and cited to support the association between insufficient sleep and diabetes mellitus. Additional publications were identified from references cited in the original articles, and the major findings were classified into different categories with narrative descriptions and also presented in tables and figures.

## 3. Results

### 3.1. Role of Sleep Disturbances in the Development of Diabetes

**Figure 2:**
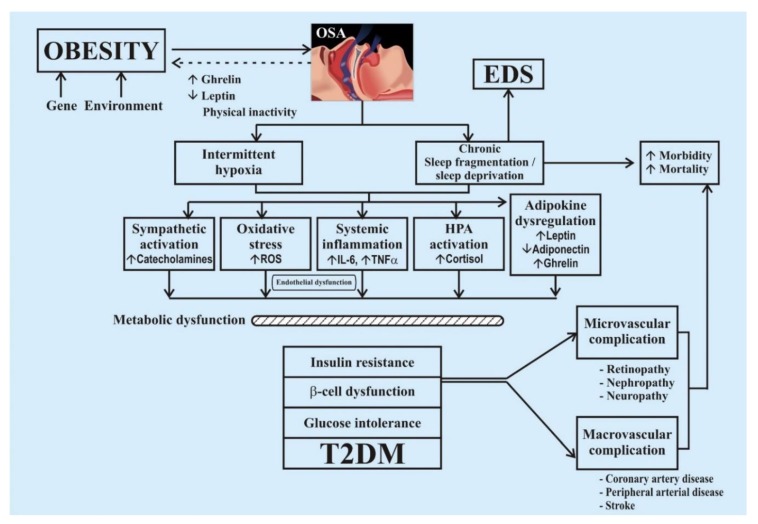
The interconnectedness and pathophysiology of obstructive sleep apnea (OSA) and diabetes. (Abbreviations: EDS: Excessive Daytime Sleepiness; HPA: Hypothalamic Pituitary Adrenal Axis; T2DM: type 2 diabetes mellitus).

The relationship between sleep disturbance and diabetes is dual-sided. Chronic sleep disturbances would elevate the risk of developing insulin resistance, while diabetes would worsen the quality of sleep. Both the qualitative and quantitative disturbances in sleep significantly increase the risk of developing diabetes. When taking into consideration quantitative aspect, it should be understood that both the short duration and long duration of sleep are associated with higher prevalence of diabetes, with the lowest risk at 7–8 h per day [10], though the underlying mechanisms and causes in both the conditions may differ [5,12,13]. OSA results in recurring episodes of hypoxemia and normoxemia as well as a drop in intra-thoracic pressure, which can lead to numerous pathophysiological conditions such as intermittent hypoxia, sleep restriction, and sleep fragmentation. These sleep disturbances result in sympathetic neural activation, systemic inflammation, oxidative stress loading, and changes in hormonal systems that lead to fat accumulation and obesity [13], as shown below (Figure 2).

Using animal models, Gozal et al. confirmed that intermittent hypoxia causes insulin resistance (IR) to deteriorate [14,15]. Several reports have demonstrated that diabetes affects central respiratory control, thereby promoting OSA. These mechanisms suggest that sleep-disordered breathing and T2DM are associated, independent of aging and obesity. In Finland, Tuomilheto et al. reported that short (≤6 h) or long (≥8 h) sleep duration is related to an increased risk of T2DM in middle-aged women [16].

### 3.2. Sleep Duration and Risk of Developing Diabetes

It is evident from various studies that both the extended hours of sleep and shorter length are related to higher risk of diabetes. Yaggi et al. [17] analyzed data from the Massachusetts Male Aging Study which included the men who were found to be borderline diabetic between the years 1987–1989, and they followed them until 2004. Men were divided into groups according to the duration of sleep: Those who slept less than 5 h, 6 h, 7 h, 8 h, and those who slept more than 8 h. They calculated the relative risk of developing diabetes. The study demonstrated that those who slept for less than 5 h were at twice as high risk of developing diabetes, while those who slept more than 8 h were at thrice as high risk of developing diabetes in comparison to those who slept for 7 h a day. Furthermore, the risk did not change when investigators considered the presence of other factors like smoking, age, education, waist circumference, and hypertension. Further study did show the relation between sleep deprivation or oversleeping and testosterone levels in men, thus suggesting the role of hormones. A study conducted in Taiwan found that a short sleep duration was associated with a higher prevalence of diabetes [18]. Another study among the Taiwanese population found that both short and long sleep durations were independently associated with newly diagnosed diabetes [19]. A study by Beihl et al. on sleep duration and type 2 diabetes in a multiethnic cohort (non-Hispanic Whites and Hispanics) found that short sleep is as an independent risk factor for type 2 diabetes in both groups [20]. A meta-analysis to assess the dose-response relationship between sleep duration and risk of type 2 diabetes concluded that the lowest risk for T2DM is among people who get 7–8 h sleep per day, whereas short and long sleep duration are associated with higher risk of T2DM [10]. Similar findings are shared by Gottlieb et al., who reported that sleep durations of short (≤6 h) or long (≥9 h) are associated with increased prevalence of DM and impaired glucose tolerance [21].

In another study, Mallon et al. studied the relationship between sleep complaints and duration of sleep with diabetes in a 12-year cohort study among Swedish population. The study randomly sampled 2663 individuals aged 5–65 years. They gave the m questions about sleep complaints, sleep duration, and other factors like depression, socio-demographic characteristics, and other comorbidities. The study demonstrated that men who slept less were at 2.8 times greater risk of developing diabetes, those who had difficulty falling asleep has almost 5 times higher risk of diabetes, and those with difficulty in maintaining sleep had 4.8 times greater risk. The study also revealed that sleep duration and quality were not much associated with the development of new diabetes in women [11]. Hence, it has been seen in several reviews and meta-analyses that both the longer duration and shorter durations are linked to a higher prevalence of diabetes. It is especially true for men, as women display a lower relationship between sleep duration and the development of diabetes [22]. In women, Facco et al. found that both shorter sleep duration and a later midpoint of sleep—independent of sleep duration—were associated with an increased risk of gestational diabetes [23]. A review conducted by Larcher et al. reported that prospective studies have demonstrated a significant risk of developing T2DM in patients having either short sleep duration or long sleep duration, which was also supported by two meta-analyses conducted from these prospective studies [24]. The other important studies showing the relation between sleep deprivation and diabetes mellitus are listed in Table 1.

### 3.3. The Relationship Between Sleep Quality and Diabetes: The Role of Sleep Apnea

Sleep apnea needs separate attention for its link with diabetes, due to an increasingly high prevalence in various demographic groups, especially in men. In the Wisconsin Sleep Cohort [42] full polysomnography was used to characterize sleep apnea in 1387 individuals, while diabetes was diagnosed either based on a physician’s diagnosis or fasting glucose above 126 mg/dl. The study demonstrated the increase in the prevalence of diabetes related to the severity of sleep apnea. Thus, the prevalence of diabetes was almost 15% in those with an apnea-hypopnea index of 15 or above. For comparison, the prevalence of diabetes was less than 3% in those with an apnea-hypopnea index of 5 or below. Thus, this study not only clearly demonstrated the links between diabetes and sleep apnea, it also confirmed the relationship between diabetes and severity of sleep apnea. When the risk of developing diabetes in four years was adjusted by taking age and sex into consideration, it was found that moderate to severe apnea increased the risk by 1.62 times. Many studies have concluded that most of the T2DM patients also suffer from OSA, which is a major risk factor for cardiovascular diseases (CVDs) and increased mortality. The interactions among the rising epidemics of obesity, OSA, and T2DM involve complex multiple pathways has serious public health implications [43]. The epidemiologic and clinical data analysis done by Zizi et al. concluded that OSA is involved in the pathogenesis of altered glucose metabolism and suggested there is an increased risk of developing T2DM resulting from curtailed sleep duration [5]. Another study to evaluate sleep duration and quality in relation to glycemic control in patients with T2DM done by Trento et al. suggested that T2DM is associated with sleep disruptions even in the absence of complications or obesity [44]. Sleep-disordered breathing (SDB) is pathophysiologically related to impaired glucose homeostasis, and that Continuous Positive Airway Pressure (CPAP) can be an important therapeutic approach for diabetic patients with SDB [45]. A review by Muraki et al. emphasized that OSA patients are more likely than non-OSA populations to develop T2DM, while more than half of T2DM patients suffer from OSA. Hence, it is very important that clinicians should take CPAP and health-related behaviors into consideration when treating T2DM and/OSA [46].

Both the Sleep Heart Health Studies (SHHS) and Atherosclerosis Risk in Communications (ARIC) study confirmed the positive association between OSA severity and T2DM during the follow-up over 12 years [47]. Another aspect of OSA—habitual snoring—has an impact on glucose metabolism in both patients with and without T2DM, as supported by various studies [43]. A 10-year follow-up study of nurses between 40–65 years found that regular snoring was independently associated with a twofold increased risk of developing T2DM [48].

Another common form of sleep disturbance is the lack of sleep hygiene and a shift in circadian rhythm due to sleep restriction owing to various reasons, such as shift work. It has been demonstrated that those who stay awake during the night are at higher risk of developing diabetes when compared with day workers. A review reported by Chattu et al. highlighted the impacts of insufficient sleep syndrome, which can lead to a range of health issues including diabetes mellitus [49]. A misalignment of the circadian rhythm has been shown to promote insulin resistance [50]. A study by Dawson et al. concluded that sleeping glucose levels decrease and are more stable after patients with type 2 diabetes and OSA are treated with CPAP [51]. It was also reported by Pallayova et al. that continuous glucose monitoring in severe sleep apnea diabetic patients before and during CPAP therapy showed significant reduction of nocturnal glucose variability and improved overnight glucose control on CPAP [52]. A study done by Knutson et al. among African Americans found the evidence linking sleep loss to increased diabetes risk. The study also reported that sleep duration and quality were significant predictors of HbA1_C_ [36]. Pamidi et al. reported that, though there is evidence to support the strong association between OSA, insulin resistance, and glucose intolerance, a causal link remains to be determined [53].

### 3.4. Diabetes and Prevalence of Sleep Disorders in Individuals Living with Diabetes

It is well demonstrated that those with sleep disorders are at higher risk of developing diabetes. However, the picture would be incomplete without considering the prevalence of sleep disorders in those with diabetes. Foster et al. [54] studied the prevalence of sleep apnea in the obese population living with type 2 diabetes. They performed polysomnography on 306 participants. Sleep apnea was diagnosed in 86% of individuals, thus confirming the relationship between sleep disorders and diabetes. In the study, 30.5% of the participants had moderate sleep apnea with an apnea-hypopnea index between 15–30, and 22.6% were diagnosed with severe sleep apnea with an apnea-hypopnea index of above 30. They also noted that the severity of sleep apnea in people with diabetes was related to waist circumference. This study confirmed the much higher prevalence of sleep apnea in those with obesity and diabetes; the study also demonstrated that sleep apnea tends to be more severe when coexisting with diabetes and obesity. A study done in Japan to assess the relationship between sleep duration and untreated diabetes in Japanese men found that short sleep duration was significantly associated with untreated diabetes in both non-obese and obese men [28]. Mahmood et al. reported that there is a statistically significant relationship between rapid eye movement (REM)-related OSA and T2DM [55]. T2DM might induce sleep apnea, but OSA facilitates the development of neuropathy. The prevalence of sleep-disordered breathing is increased in patients with diabetes and autonomic neuropathy (AN) [56]. Type 1 diabetes is comparatively uncommon, with around 5–6% of those diagnosed with diabetes having type 1 diabetes [57]. Type 1 diabetes is very different from T2DM, as it is a disease that is usually diagnosed at a young age; most of those diagnosed are lean, and dietary habits or lifestyle has far muted role in disease development. However, studies do confirm the sleep disturbances in those with type 1 diabetes, though the underlying mechanism seems to be entirely different. In type 1 diabetes, there is a clear link between neuropathy and sleep disturbances; thus, sleep apnea may be either obstructive or central to type 1 diabetes [58]. Rajan et al. reviewed the links and bidirectional associations between OSA and T2DM and found that there is a high prevalence of insulin resistance and T2DM in patients with OSA. An even higher prevalence of OSA has been documented in those patients with T2DM who are obese [59]. Zhu et al. conducted another longitudinal study among 64 patients with T2DM in the US to see the relationship between sleep and self-care found; they found that subjective sleep quality, diabetes distress, and daytime sleepiness were strong predictors of self-care when objective sleep parameters were used. The study suggested that subjective sleep quality and objective nocturnal awakenings might affect self-care in older patients with T2DM [60].

## 4. Discussion

### 4.1. Mechanism of Development of Diabetes in Sleep Disorder

Sleep disorders lead to insulin resistance and beta-cell dysfunction through various pathways [61]. Hypoxia, sleep fragmentations, and activation of the sympathetic nervous system are some of the pathways that play a significant role in the development of T2DM in those with sleep disorders [62,63,64,65,66,67,68]. Sleep fragmentation results in elevated sympathetic activity and a higher level of inflammation [2]. Sleep fragmentation may also result in obesity. It is also possible that sleep fragmentation may lead to an adipose tissue inflammation that is NADPH oxidase-2 (NOX2) mediated [6]. A review by Kent et al. concluded that intermittent hypoxia (IH) and sleep deprivation likely play crucial roles in the pathogenesis of glucose metabolic dysfunction in OSA, thus contributing to various pathways synergistic with obesity [69]. The evidence from epidemiological and experimental studies concluded that OSA results in glucose intolerance, which leads to T2DM [70,71,72,73]. Sleep deprivation has been associated with multiple physiological changes including increased cortisol and ghrelin levels, decreased leptin levels, and impaired glucose metabolism [74]. A recent study reported by McLain confirms that sleep fragmentation delays wound healing in type 2 diabetes. In this animal experiment model, they found that sleep fragmentation caused a substantial rise in wound levels of TNF-α mRNA in the mice, and there was delayed wound healing in obese and diabetic mice as the altered leptinergic signals and inflammatory proteins result in delayed wound healing [75].

When a person is asleep, the parasympathetic nervous system is predominant, resulting in a slowdown of heart rate, blood pressure, respiration rate, gut movement, other bodily functions, body temperature, and basal metabolism. However, if sleep is disturbed too often, this predominance of the parasympathetic nervous system does not occur, and the sympathetic tone is elevated. It results in a higher load on the circulatory system, a higher rate of basal metabolism, a higher level of stress hormones, and, finally, a higher hazard of developing insulin resistance or diabetes [76]. Barone et al. highlighted that the association between sleep and diabetes may be described as a vicious circle, where sleep disorders favor the development of T2DM or aggravate both types of diabetes. On the other hand, diabetes itself when accompanied with poor metabolic control is mostly followed by sleep disorders [77]. A study by McMullan et al. found that lower melatonin secretion was independently associated with a higher risk of developing T2DM [78].

Hypoxia is another important mechanism behind the development of insulin resistance [79]. Hypoxia in sleep apnea differs because it is intermittent. Thus, there are periods of low oxygen perfusion followed by compensatory high oxygen perfusion. It disturbs the normal metabolism, resulting in the elevated formation of reactive oxygen, which is damaging to various cells. Another proposed pathway of insulin resistance in intermittent hypoxia is through the inflammation of adipose tissues. In mouse models, it has been demonstrated that intermittent hypoxia leads to downregulation of insulin receptors, lower uptake of glucose by adipocytes, and higher levels of pro-inflammatory markers [80].

OSA was found to be associated with metabolic syndrome independent of obesity predominantly due to increased triglycerides, glucose and Epworth score values but not insulin resistance [81]. The Epworth Sleepiness Scale has eight items that measure subjective sleepiness, and a value above 10 indicates the presence of excessive daytime sleepiness (EDS). In fact, chronic intermittent hypoxia has been proposed as the single most crucial factor in the development of insulin resistance in those with sleep apnea. It seems that intermittent hypoxia may directly cause beta cells dysfunction, and it may lead to changes in liver enzymes and liver functioning, thus affecting the glucose homeostasis [82]. Chronic intermittent hypoxia may also be related to changes in skeletal muscles and glucose uptake [64]. The Sleep Heart Study found that sleep-disordered breathing (SDB) is independently associated with glucose intolerance and IR, which may lead to T2DM [83]. Spiegel et al. reported that chronic sleep loss—whether behavioral or sleep disorder related—may represent a novel risk factor for weight gain, IR, and T2DM [84]. Though the relationship between long sleep duration and a higher risk of diabetes is well established, no plausible explanation has been found. It seems that IR is probably caused by sleep disturbances and altered sleep quality in prolonged duration of sleep, though it remains the subject of further investigations [12].

### 4.2. Aspects of Insufficient Sleep (Quality and Quantity) Leading to Diabetes

If sleep disruption—qualitative or quantitative—may lead to obesity, insulin resistance, and development of diabetes, then it is entirely possible that simple, low-cost methods of sleep correction may help to prevent diabetes in a vast number of cases. Though there has been limited research in this direction, some of the research seems to support the idea of the positive effect of adequate sleep on insulin sensitivity. Sleep deprivation and sleep disorders contribute to pathophysiological changes associated with the development of T2DM, and, among diabetes, sleep deprivation contributes to elevations of HbA1c [85]. A recently published Norwegian study concluded that, along with obesity and hypertension, insomnia was the major modifiable factor associated with T2DM [86]. Aurora et al. reported the bidirectional link between OSA and T2DM and concluded that early identification of obstructive sleep apnea in patients with metabolic dysfunction—including type 2 diabetes—and assessment for metabolic abnormalities in those with obstructive sleep apnea could reduce cardiovascular disease risk and improve the quality of life of patients [87].

A study by Shankar et al. on perceived insufficient rest included in the Behavioral Risk Factor Surveillance System (BRFSS) of the USA found that perceived insufficient rest/sleep are found to be independently associated with CVDs, diabetes mellitus and obesity [88]. Another Australian prospective study and meta-analysis aimed to determine whether short sleep duration predicts future CVD or type 2 diabetes and found that the risk of incident type 2 diabetes was significantly increased in those with <6 h versus 7 h of sleep. A meta-analysis of ten prospective studies including 447,124 participants also confirmed an association (1.33) between short sleep and incident diabetes (1.20–1.48). Obtaining less than 6 h of sleep each night (compared to 7 h) may increase type 2 diabetes risk by approximately 30% [89].

Thus, in one of the interventions, obese volunteers were asked to increase their sleep duration by just one hour; they were intermittently monitored for sleep quality and fasting glucose. The study was continued for 40 days. At the end of the research, insulin sensitivity and fasting glucose were improved just through sleep prescription, thus demonstrating the value of this low-tech and low-cost approach to diabetes preventions [90]. The review on OSA and diabetes reported by Tahrani et al. concluded that OSA is associated with insulin resistance and β-cell dysfunction independent of obesity. OSA is associated with HbA1c and vascular complications in patients with T2DM [91].

There have been a number of studies regarding the beneficial effect of treating chronic intermittent hypoxia in those diagnosed with obstructive sleep apnea. One of the most effective treatment approaches is the use of CPAP. A recent study reported by Malik et al. concluded that the treatment of OSA with CPAP reduces the HbA1c in a significant number of diabetics [92]. In the meta-analysis of various random clinical trials, CPAP has been demonstrated to provide significant benefit in reducing insulin resistance and thus decreasing the risk of developing diabetes. Conversely, it should be understood that good glycemic control in diabetes would result in better sleep quality and decrease in REM sleep latency, thus serving to improve the quality of life and reducing the risk of other adverse effects of sleep deprivation [93]. Overall, improving sleep duration and quality is a potential tool to counteract the epidemics of obesity and diabetes [94]. A prospective cohort study done in Japan to investigate the effect of self-reported sleep duration and sleep quality on the risk of developing diabetes concluded that medium and high frequencies of difficulty initiating sleep are associated with higher risks of diabetes mellitus in relatively healthy Asian workers [34]. Excessive long and short duration of sleep is associated with type 2 diabetes, as we have seen it from numerous epidemiological studies. A recent study from Japan investigated the link between sleep quality and diabetes among 3249 diabetic patients (<20 years) using the Pittsburgh Sleep Quality Index (PSQI); these patients were found to have poor subjective sleep quality due to increased sleep latency and a shorter duration of sleep [95]. The short duration of sleep is associated with increased incidence of T2DM as well as poor metabolic control in both type 1 and type 2 DM [26]. Though the purpose of this review is not to address other therapeutic modalities for the treatment of diabetes, one should bear in mind that for a holistic approach, weight loss (e.g., dietary and lifestyle modifications) and surgical options are available. It has been proposed that improving sleep quality, treating sleep disorders, and optimizing sleep duration could be used as a regimen to indirectly promote glycemic control [36,77]. A recently published review by Chattu et al. emphasized that sleep quality assessments can be important early risk indicators thereby reducing the incidence of a wide spectrum of morbidities [96]. Another review published in Lancet by Tan et al. [97] critically analyzed various common treatments for insomnia and OSA on both sleep and glucose metabolism in patients with type 2 diabetes. The various therapeutic interventions or treatments included pharmaceutical sleep aids (e.g., benzodiazepine receptor agonists, melatonin), cognitive behavioral therapy for insomnia (CBT-I), CPAP for OSA, and lifestyle interventions, (e.g., aerobic exercise training and dietary interventions).

### 4.3. Limitations

The review considered mostly the articles which are published after the year 2000 (most of the searches showed articles published after 2000) and did not looked into the individual historical studies done before; however, the interpretation of the meta-analysis and systematic reviews of those prior studies was discussed. Due to the availability of exhaustive literature in various languages on this topic, the authors limited their search only to English language and full text articles. Some of the conference proceedings and other presentations were not considered for this review, as decided earlier as per the inclusion criteria.

## 5. Conclusions

In conclusion, it can be said that qualitative and quantitative sleep disorders can increase the risk of insulin resistance and diabetes. A sleep disorder can also increase the risk of complications related to diabetes. The relationship between sleep and diabetes is bi-directional, meaning that well-managed diabetes would result in better sleep quality, too. There are certain limitations to what we know about the relationship between sleep and diabetes. First, the underlying mechanism is not wholly understood. Second, most of the studies looking at the link between the conditions have focused on type 2 diabetes. Thus, there is little information about the role of sleep disorders in type 1 diabetes. Finally, it may be wise to say that the importance of sleep prescription and sleep hygiene has not been fully realized in diabetes prevention.

## Figures and Tables

**Table 1:** Evidence on sleep deprivation and diabetes mellitus from various studies.

No.	Reference	Country	Year	Target Population	Type of Study	Sample Description (Sample (N); Males (M); Females (F); Age Range (AR))	Study Duration	Inference/Major Findings
1	Seixas et al. [25]	USA	2018	General population	National Health Interview Survey with face-face interviews and questionnaires	N = 236,406; M = 45%; F = 55%; AR = 18–85 years	10 years (survey data) (2004–2013)	Both short sleep and long sleep were associated with diabetes mellitus. Among cancer survivors, short sleep was associated with higher self-reported diabetes.
2	Matsumoto et al. [26]	Japan	2018	Community participants	Cross-sectional	N = 7051	1 year	Sleep-disordered breathing (SDB) was associated with a higher risk of diabetes in premenopausal women and postmenopausal women but not in men. SDB and obesity were independently associated with diabetes.
3	Facco et al. [27]	USA	2017	Nulliparous women during pregnancy (16 0/7 and 21 6/7 weeks’ gestation)	Prospective Cohort Study	N = 782; F ≥ 18 years	<year till the delivery	Short sleep duration (<7 h) and a later sleep midpoint are proven to increase the risk of gestational diabetes.
4	Lin et al. [18]	Taiwan	2016	Secondary data from the Nutrition and Health Survey	Cross-sectional	N = 1533; M = 733; F = 800; AR = 19–64 years	3 years (2005–2008)	Risk of diabetes among 19–44 years with ≤5 h of sleep was 5.24-fold higher than who reported 7–8.9 h of sleep at night.
5	Kachi et al. [28]	Japan	2012	Routine Health Assessments data	Cross-sectional	N = 20,744; M = 20,744; AR = 30–64 years	2003–2007	Men sleeping for ≤5 h and ≥8 h were more likely to have untreated diabetes compared to those who had 7 h sleep.
6	Qui et al. [29]	USA	2010	Pregnant women (<20 weeks gestation)	Prospective Cohort	N = 1290; F = 1290; AR ≥ 18 years	2003–2006	Short sleep duration is strongly associated with glucose intolerance and gestational diabetes.
7	Facco et al. [23]	USA	2010	Nulliparous women in pregnancy (6–20 weeks of gestation)	Prospective cohort study	N = 189; F = 189; AR ≥ 18 years	2007–2008 (16 months)	Short sleepers had glucose intolerance during pregnancy.
8	Rafalson et al. [30]	USA	2010	Participants with cardiovascular disease but no history of diabetes	Nested Case-Control Study	N = 1455; AR = 35–79 years	6 years (1996–2001)	Short sleepers had an increased risk of impaired fasting glucose due to insulin resistance.
9	Xu et al. [31]	USA	2010	164,399 without diabetes and 10,143 participants with diabetes diagnosed after 2000	Prospective study	N = 174,542; AR = 50–71 years	2000–2006	Day napping and a short duration of sleep showed a positive association with diabetes.
10	Hall et al. [32]	USA	2008	Adult Health and Behavior Project Registry	Cross-sectional Community-based cohort study	N = 1214; M = 568; F = 646 AR = 30–54 years	2006	Short and long sleepers were at 45% increased risk of having metabolic syndrome compared to those with 7–8 h of sleep
11	Choi et al. [33]	South Korea	2008	Korean Health and Nutrition Survey	Cross-sectional	N = 4222; M = 1822; F = 2400; AR ≥ 20 years	1 year	Short and long sleep durations - increase the risk of metabolic syndrome compared to those with 7 hrs of sleep.
12	Tuomilehto et al. [16]	Finland	2008	FIN-D2D survey is a population-based survey	Population-based cross sectional study	N = 2800; M = 1366; F = 1434; AR = 45–74 years	2 years (2004–2005)	Short (<6 h) or long (>8 h) sleep duration increased the risk of type 2 diabetes in middle-aged women but not in men.
13	Hayashino et al. [34]	Japan	2007	High-risk and Population Strategy for Occupational Health Promotion Study	Cohort study	N = 6509; AR = 19–69 years	6 years (1999–2004)	Among healthy adult subjects, the risk of diabetes was linked to difficulty initiating sleep.
14	Gangwisch et al. [12]	USA	2007	National Health and Nutrition Examination Survey	Multivariate longitudinal analyses	N = 8992 AR = 32–86 years	10 years (1982–1992)	Short sleep is a risk factor for diabetes. The association between long sleep duration and diabetes due to some unmeasured confounder like poor quality of sleep.
15	Chaput et al. [35]	Canada	2007	Quebec Family Study	Cross-sectional	N = 740; M = 323; F = 417; AR = 21–64 years	3 years (1989–2001)	Sleep of <6 h resulted in impaired glucose tolerance (IGT). Short- and long-duration sleep times are associated with T2DM/IGT in adults.
16	Yaggi et al. [17]	USA	2006	Massachusetts Male Aging Study without diabetes	Cohort study	N = 1709 (1139); AR = 40–70 years	18 years (1987–2004)	Short and long sleep durations are proved to increase the risk of T2DM.
17	Knutson et al. [36]	USA	2006	Volunteers with type 2 diabetes	Cross-sectional	N = 161; M = 42; F = 119; AR = 57 years (average)	2006	Both sleep duration and quality are significant predictors of HbA1c, which is crucial for glycemic control.
18	Meisinger et al. [37]	Germany	2005	MONICA Augsburg surveys—general population	Cross-sectional	N = 8269; M = 4140; F = 4129; AR = 25–74 years	12 years (1984–1995)	Difficulty maintaining sleep was associated with an increased risk of type 2 diabetes in men and women.
19	Bjorkelund et al. [38]	Sweden	2005	Women	Prospective study	N = 661; F = 661	32 years	Sleep problems and developing diabetes were not linked in this 32-year follow-up study of middle-aged women. Obesity, known to cause increased risk of T2DM, was associated with sleep problems.
20	Mallon et al. [11]	Sweden	2005	A random sample of 2663 subjects	Cohort study	N = 2663 (1170); M = 550; F = 620; AR = 45–65 years	12 years (1983–1995)	Difficulty in sleep maintenance and short sleep duration increases T2DM in men.
21	Gottleib et al. [21]	USA	2005	Sleep Heart Health Study	Cross-sectional	N = 1486; M = 722; F = 764; AR = 53–93 years	1995–1998	Subjects sleeping 6 h or less had adjusted odds ratio for Diabetes of 2.51 and 1.66, respectively. Sleep duration <6 h or >9 h is associated with an increased prevalence of DM and IGT.
22	Nilsson et al. [39]	Sweden	2004	Prospective population-based study	Cohort study	N = 6599	14.8 ± 2.4 years	Sleep disturbances are proven to increase the risk of T2DM.
23	Kawakami et al. [40]	Japan	2004	Male employees of the company	Prospective study	N = 2649; M = 2649	8 years (1984–1992)	Sleep disturbances resulted in 2–3 times increase in the risk of T2DM.
24	Ayas et al. [41]	USA	2003	Nurses Health Study (without diabetes)	Cohort study	N = 70,026; AR = 40–65 years	10 years	Sleep restriction may be an independent risk factor for developing T2DM.
